# Supplementary material for: Gene Expression Analysis Reveals Potential Regulatory Factors Response to Temperature Stress in Bemisia tabaci Mediterranean
Source: Genes (Basel). 2023 Apr 29;14(5):1013. doi: 10.3390/genes14051013 (PMC10218290; doi:10.3390/genes14051013)
Supplement: Supplementary file 1 [file genes-14-01013-s001.zip › genes-2318853-supplementary.pdf]

genes

MDPI

Article

Gene Expression Analysis Reveals Potential Response Factors to Temperature in *Bemisia tabaci* Mediterranean

Xiao-Na Shen[1, 2], Xiao-Di Wang[2], Fang-Hao Wan[2, 3], Zhi-Chuang Lü[2,*], Wan-Xue Liu[2],

[1] Department of Basic Medicine, Changzhi Medical College, Changzhi, 046000, China;
[2] State Key Laboratory for Biology of Plant Diseases and Insect Pests, Institute of Plant Protection, Chinese Academy of Agricultural Sciences, Beijing 100193, PR China;
[3] Agricultural Genome Institute at Shenzhen, Chinese Academy of Agricultural Sciences, Shenzhen 518120, PR China;
* Correspondence: lvzhichuang@caas.cn; Tel./Fax: +86 10 82109572

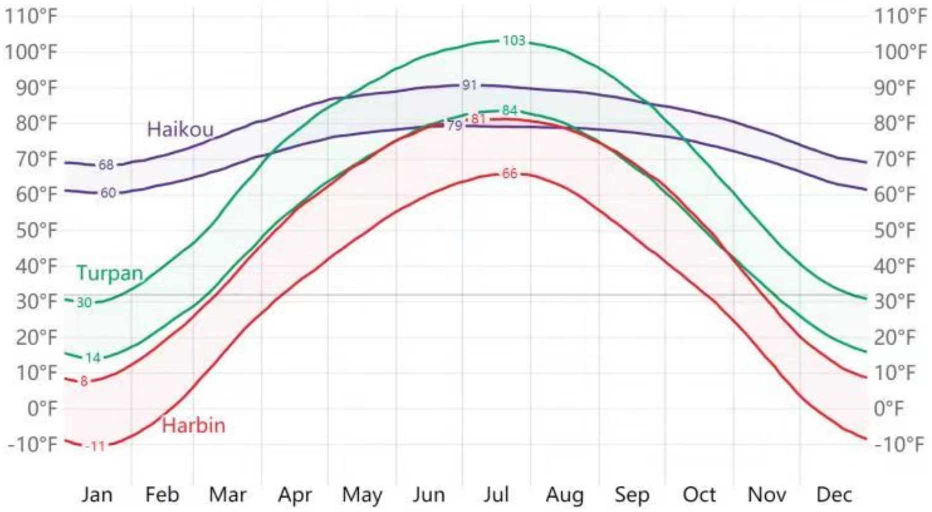

Supplementary Figure S1.: The daily average high and low air temperatures at two meters above the ground (© WeatherSpark.com).

Citation: To be added by editorial staff during production.

Academic Editor: Firstname Lastname

Received: date
Revised: date
Accepted: date
Published: date

Copyright: © 2023 by the authors. Submitted for possible open access publication under the terms and conditions of the Creative Commons Attribution (CC BY) license (https://creativecommons.org/licenses/by/4.0/).

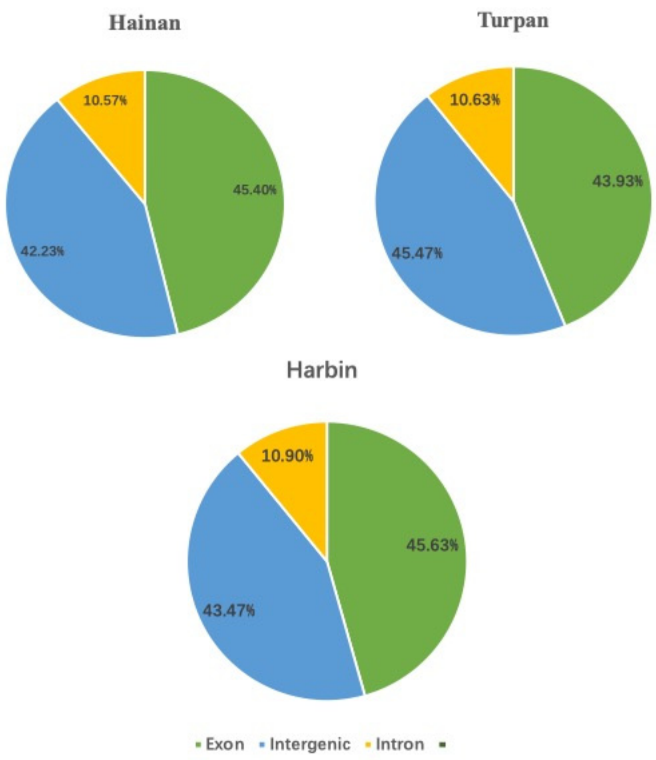

Supplementary Figure S2. The distribution of reads mapped to reference genome from three *Bemisia tabaci* geographic populations

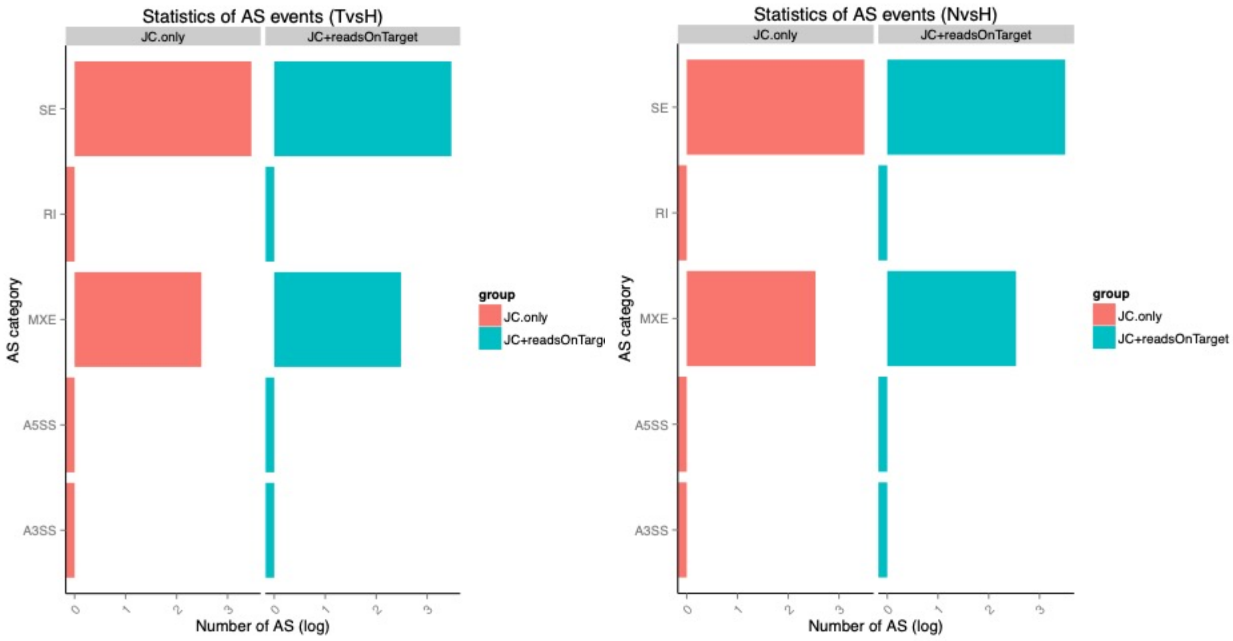

Supplementary Figure S3. Classification and Counting of Alternative splicing Events from two comparisons (T: Turpan; H: Harbin; N: Hainan)

* SE：Skipped exon; MXE: Mutually exclusive exon; A5SS: Alternative 5' splice site; A3SS: Alternative 3' splice site; RI: Retained intron

Supplementary Table S1 Summary of the quality of data output

Sample name	Raw reads	Clean reads	clean bases	Error rate(%)	Q20(%)	Q30(%)	GC content(%)
T_1	42000720	39894888	5.98G	0.02	95.55	89.44	37.15
T_2	44783106	42276006	6.34G	0.02	94.9	88.34	36.64
T_3	46750216	44541656	6.68G	0.02	95.51	89.23	37.25
H_1	46525472	44139430	6.62G	0.02	95.62	89.66	37.59
H_2	52915098	49816812	7.47G	0.02	95.28	88.73	37.59
H_3	59508372	56266916	8.44G	0.02	95.29	88.79	37.4
N_1	49765444	47823190	7.17G	0.02	95.74	89.75	38.41
N_2	44400592	41869522	6.28G	0.02	95.85	90.17	37.57
N_3	45833748	44061888	6.61G	0.02	95.68	89.83	36.48

genes

MDPI

Supplementary Table S2 List of reads and reference genome alignment

**Sample name**	**T_1**	**T_2**	**T_3**	**H_1**	**H_2**	**H_3**	**N_1**	**N_2**	**N_3**
Total reads	39894888	42276006	44541656	44139430	49816812	56266916	47823190	41869522	44061888
Total mapped	28626392 (71.75%)	28610618 (67.68%)	32632565 (73.26%)	31263253 (70.83%)	36536167 (73.34%)	39056824 (69.41%)	35329405 (73.88%)	30139913 (71.99%)	30301158 (68.77%)
Multiple mapped	3686583 (9.24%)	3812385 (9.02%)	4289457 (9.63%)	4302940 (9.75%)	4926232 (9.89%)	5439613 (9.67%)	4676941 (9.78%)	4134175 (9.87%)	4141156 (9.4%)
Uniquely mapped	24939809 (62.51%)	24798233 (58.66%)	28343108 (63.63%)	26960313 (61.08%)	31609935 (63.45%)	33617211 (59.75%)	30652464 (64.1%)	26005738 (62.11%)	26160002 (59.37%)
Read-1	12708964 (31.86%)	12656388 (29.94%)	14411048 (32.35%)	13722653 (31.09%)	16106051 (32.33%)	17066394 (30.33%)	15598975 (32.62%)	13217681 (31.57%)	13326529 (30.25%)
Read-2	12230845 (30.66%)	12141845 (28.72%)	13932060 (31.28%)	13237660 (29.99%)	15503884 (31.12%)	16550817 (29.41%)	15053489 (31.48%)	12788057 (30.54%)	12833473 (29.13%)
Reads map to '+'	12451550 (31.21%)	12384769 (29.3%)	14152379 (31.77%)	13461663 (30.5%)	15793250 (31.7%)	16799527 (29.86%)	15316235 (32.03%)	12989965 (31.02%)	13064610 (29.65%)
Reads map to '-'	12488259 (31.3%)	12413464 (29.36%)	14190729 (31.86%)	13498650 (30.58%)	15816685 (31.75%)	16817684 (29.89%)	15336229 (32.07%)	13015773 (31.09%)	13095392 (29.72%)
Non-splice reads	19298666 (48.37%)	19758616 (46.74%)	21579613 (48.45%)	20405033 (46.23%)	23657048 (47.49%)	25770124 (45.8%)	22564645 (47.18%)	19814815 (47.33%)	20511928 (46.55%)
Splice reads	5641143 (14.14%)	5039617 (11.92%)	6763495 (15.18%)	6555280 (14.85%)	7952887 (15.96%)	7847087 (13.95%)	8087819 (16.91%)	6190923 (14.79%)	5648074 (12.82%)

genes MDPI

Supplementary Table S3 Classification and quantity statistics of Alternative splicing events in comparison between Turpan and Harbin

Event Type	Num Events. JC. only	Sig Events. JC. only	Num Events. JC. + reads On Target	Sig Events. JC. + readsOn Target
Skipped exon	2927	185 (107:78)	2981	195 (113:82)
Mutually exclusive exon	306	67 (34:33)	306	71 (38:33)
Alternative 5' splice site	0	0 (0:0)	0	0 (0:0)
Alternative 3' splice site	0	0 (0:0)	0	0 (0:0)
Retained intron	0	0 (0:0)	0	0 (0:0)

* Num Events. JC. only: total number of alternative splicing events detected only by junction counts; Sig Events. JC. only: total number of differential alternative splicing events detected only by Junction Counts (the number of up-regulated alternative splicing events: the number of down-regulated alternative splicing events); Num Events. JC. + reads On Target: total number of alternative splicing events detected using both junction counts and reads on target. Sig Events. JC. + reads On Target: total number of differential alternative splicing events detected using both junction counts and reads on target (the number of up-regulated alternative splicing events: the number of down-regulated alternative splicing events).

Supplementary Table S4 Classification and quantity statistics of Alternative splicing events in comparison between Hainan and Harbin

Event Type	NumEvents.JC.only	SigEvents.JC.only	NumEvents.JC.+readsOnTarget	SigEvents.JC.+readsOnTarget
Skipped exon	3159	159(76:83)	3212	168 (78:90)
Mutually exclusive exon	345	73 (35:38)	345	80 (41:39)
Alternative 5' splice site	0	0 (0:0)	0	0 (0:0)
Alternative 3' splice site	0	0 (0:0)	0	0 (0:0)
Retained intron	0	0 (0:0)	0	0 (0:0)

*Same as supplementary Table 3.

Supplementary Table S5 Distribution of differential expressed genes in GO terms enriched in biological processes, cell components, and molecular functions

**Go terms**	**NvsH**	**TvsH**	**TvsN**
Biological process	62.43%	58.44%	62.15%
Cellular component	11.29%	14.46%	11.46%
Molecular function	26.28%	27.10%	26.39%
